# Crop Agnostic Monitoring Driven by Deep Learning

**DOI:** 10.3389/fpls.2021.786702

**Published:** 2021-12-20

**Authors:** Michael Halstead, Alireza Ahmadi, Claus Smitt, Oliver Schmittmann, Chris McCool

**Affiliations:** Agricultural Robotics, Institute of Agricultural Engineering, University of Bonn, Bonn, Germany

**Keywords:** plant classification, artificial intelligence, deep learning, convolutional neural network, image segmentation, field plant observation

## Abstract

Farmers require diverse and complex information to make agronomical decisions about crop management including intervention tasks. Generally, this information is gathered by farmers traversing their fields or glasshouses which is often a time consuming and potentially expensive process. In recent years, robotic platforms have gained significant traction due to advances in artificial intelligence. However, these platforms are usually tied to one setting (such as arable farmland), or algorithms are designed for a single platform. This creates a significant gap between available technology and farmer requirements. We propose a novel field agnostic monitoring technique that is able to operate on two different robots, in arable farmland or a glasshouse (horticultural setting). Instance segmentation forms the backbone of this approach from which object location and class, object area, and yield information can be obtained. In arable farmland, our segmentation network is able to estimate crop and weed at a species level and in a glasshouse we are able to estimate the sweet pepper and their ripeness. For yield information, we introduce a novel matching criterion that removes the pixel-wise constraints of previous versions. This approach is able to accurately estimate the number of fruit (sweet pepper) in a glasshouse with a normalized absolute error of 4.7% and an *R*^2^ of 0.901 with the visual ground truth. When applied to cluttered arable farmland scenes it improves on the prior approach by 50%. Finally, a qualitative analysis shows the validity of this agnostic monitoring algorithm by supplying decision enabling information to the farmer such as the impact of a low level weeding intervention scheme.

## 1. Introduction

Agricultural robotics and automation is a rapidly developing field, driven by advances in artificial intelligence (AI). For agricultural robotics, advances in AI and robotic vision has meant that interaction with crops has been enabled, exemplified by robotic seeding (Utstumo et al., 2018), weed management (Bawden et al., 2017), and harvesting (Lehnert et al., 2017; Arad et al., 2020). To date, agricultural automation researchers have used coarse inputs such as temperature, lighting, and CO_2_ which are inputs to AI-based approaches to control the outputs (e.g., lighting and nutrients) to the crop (Hemming et al., 2019). Yet, there are obvious gains to be made by providing more frequent and finer-grained inputs about the state of the crop.

**Figure 1:**
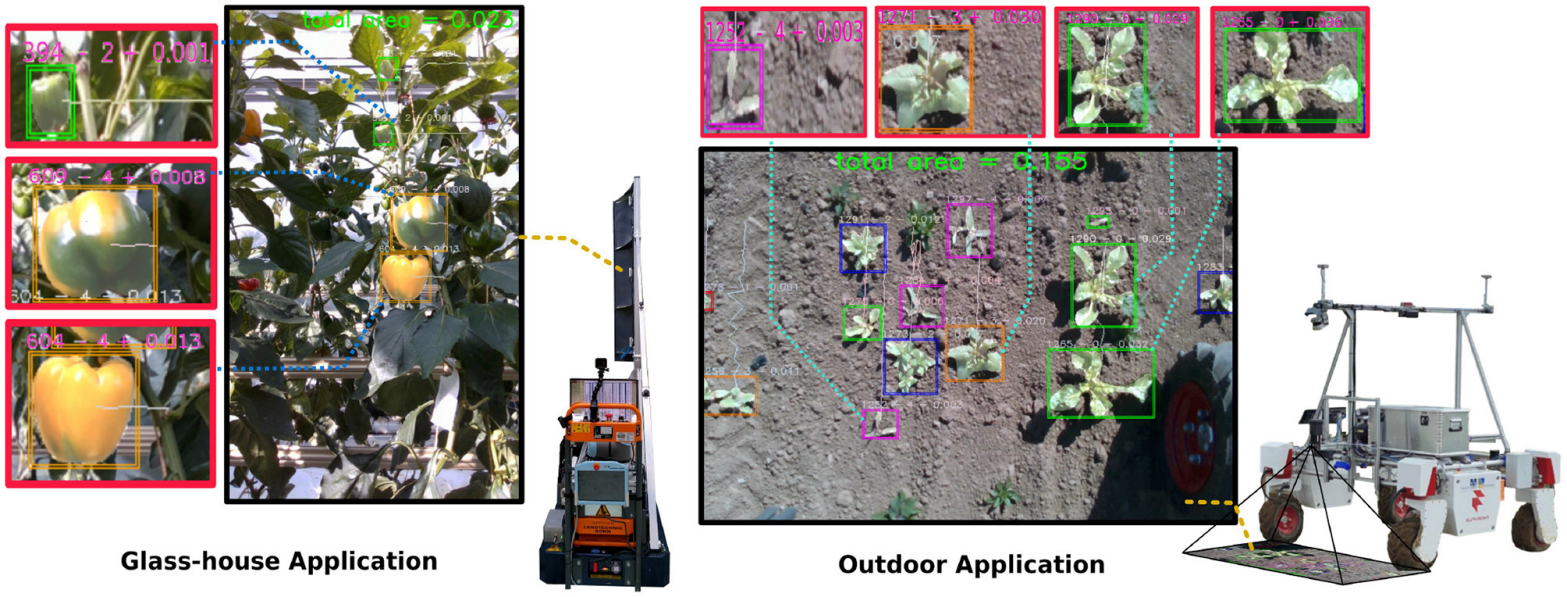
The agnostic monitoring algorithm provides up-to-date information to the farmer based on instance segmentation with ripeness or species information and area estimation. This assists in making more informed management decisions such as weeding or harvesting using a tracking-*via*-segmentation approach for yield estimation. The approach is evaluated on two robotic platforms PATHoBot **(Left)** and BonnBot-I **(Right)** which work in significantly different environments: glasshouse or arable fields. Area estimation values are in m^2^.

From the stakeholder (farmer) perspective, monitoring plants and their ecosystem is a key element to making informed management decisions. Without robotics or automation, a farmer has to physically view (traverse or spot check) their farm multiple times and pay attention to critical markers such as the number of fruit or crop, or the presence of weeds, pests, or diseases, as advised by LWK-Rheinland (2020) in Germany. Robotic platforms have the potential to both automate and enhance these observations by performing these repetitive tasks with a high degree of accuracy, as outlined in Figure 1. This high degree of accuracy comes from recent advances in robotic vision.

From a robotic monitoring, perspective platforms need to be designed to ascertain relevant information, not just all information, simplifying data collection for the end user. From a cost perspective, these monitoring platforms need to reduce the impact of labor on costs (from physical viewing) which is a key expenditure for farming operations (ABARES, 2018). As an example, in arable farming, the farm should be monitored multiple times to measure crop germination and the current growth stage, enabling decisions, such as fertilization, herbicide, fungicide, and insecticide treatments (LWK-Rheinland, 2020). Robotics provides a potential mechanism to automate the crop monitoring process, however, currently, the platforms are designed for a specific purpose or their algorithms are designed for a specific platform, limiting uptake.

From the robotics and robotic vision fields, research has progressed considerably over the past decade with a particular focus on estimating the presence or potential yield of the crop. In the previous decade, specialized vision algorithms were developed for particular crops, such as grapes (Nuske et al., 2011, 2014), apples (Wang et al., 2012), tomatoes (Yamamoto et al., 2014), and almonds (Hung et al., 2013). These methods relied on traditional computer vision approaches and hand crafted features were defined for each crop. However, more recently, deep learning approaches such as DeepFruits have been proposed by Sa et al. (2016). Such approaches are amenable to being deployed to multiple crops. These, more generalizable, approaches are integral if we are to perform automated monitoring without human intervention.

More recently, robots that better integrate vision systems, deep learning, and robotics have been proposed. These robots can perform not only a specific action, or intervention, but also repeatedly estimate the state of the field. An example of this is PATHoBot (Smitt et al., 2021) which aims to combine advances in deep learning with robot (platform) information to provide a more robust crop monitoring approach. In this approach, the camera parameters and wheel odometry were utilized to refine a tracking algorithm based on deep learned semantic masks.

In this article, we present a platform agnostic algorithm for monitoring the state of arable farmland (crop/weed management) and glasshouses (crop management). We propose to improve the AI and tracking components outlined in Halstead et al. (2018, 2020) and evaluate it on both PATHoBot (Smitt et al., 2021) (glasshouse) and BonnBot-I (Ahmadi et al., 2021a) (arable farmland). This approach monitors the crops in the various environments and assists the farmer in making informed management decisions. Our technique is evaluated on two robots across two vastly different environments and demonstrated the potential to

- Perform generalized plant segmentation in arable farmland and provide more fine-grained classification labels;
- Sweet pepper segmentation and ripeness classification using the same network as arable farmland;
- Incorporation of area estimation calculations of both the crop (sugar beet (SB), and sweet pepper) and weeds where appropriate;
- Tracking-*via*-segmentation matching criterion evaluation and novel approach to alleviate cluttering issues; and,
- Fuse all techniques into a single monitoring technique for agriculture.

The paper is organized as follows: section 2 outlines the prior work in this field; the materials being used are described in section 3; the methods are outlined in section 4; we display and discuss our results in section 5; and finally we conclude the paper in section 6.

## 2. Related Work

Crop monitoring is an important facet of any farm, from arable farming (wheat, corn, SB, etc.) to horticulture (apples, tomatoes, sweet peppers, etc.). An example in the context of horticultural farming is knowledge about the state of the field, such as the number and quality of fruit by Halstead et al. (2018) or estimating the final yield by Nuske et al. (2011). For arable farming the presence and number of weeds are important, and several platforms have been designed to manage them (Bakker et al., 2010; Peruzzi et al., 2017).

From a monitoring perspective, weed management highlights the need for the entire process to be strategic, from monitoring to intervention. This is because the indiscriminate removal of weeds can have a negative impact on both soil and crop health (Blaix et al., 2018). Therefore, informed monitoring of crop can help improve the bio-diversity by using selective weeding protocols (Blaix et al., 2018; Adeux et al., 2019). Using a well-designed monitoring platform can ensure that the crop can be positively impacted by weeds which can increase pollinators when the herbicide is decreased (Raven and Wagner, 2021). Arable farmland is just one area where careful monitoring paradigms can increase the health of the crop through perceptive information. The key enabling technology for these are agricultural robots and their associated robotic vision algorithms. Below, we briefly describe relevant prior work in terms of robotics in agriculture, the vision systems to enable robotic systems and the object tracking systems that enable them to summarize the content of the field (a key element of this work).

### 2.1. Robotics in Agriculture

Robotics and robotic vision have long been thought to provide a way to perform regular autonomous crop monitoring. This includes increasing the frequency, both spatially and temporally, of field monitoring to improve management. Several robotic platforms have been proposed with either crop monitoring (Smitt et al., 2021) or intervention (Utstumo et al., 2018) (performing an action on the crop) in mind. Yet, in agriculture (arable and horticultural farming), the robotic platform and their robotic vision algorithms have been inexorably intertwined.

From an arable farming perspective, the most prevalent robotic platforms deal with weed management. Slaughter et al. (2008) produced an early study into various weeding techniques and outlined the negative impact of herbicides. Bakker et al. (2010) developed a weeding platform that was able to operate inside crop-row fields, particularly between the crop. The primary goal of their technique was to replace manual weeding. A review conducted by Peruzzi et al. (2017) outlined many of the possible weeding techniques, along with automated versions, for crop-row fields. While this article did not concentrate specifically on robotic platforms, it does provide a solid overview into weed management techniques not dependent on herbicide. From an automated weeding perspective, AgBot II (Bawden et al., 2017) was able to designate the type of intervention, mechanical or chemical, based on the type of weed and a vision based detection routine. To reduce herbicide use, Utstumo et al. (2018) used machine learning (ML) and computer vision to control a drop-on-demand weeder. Their technique also allowed for more powerful herbicide use as crop health was assured with their spraying approach.

From a horticultural farming perspective, the harvesting of fruit has been a commonly tackled issue. Lehnert et al. (2017) built a sweet pepper harvesting robot that was able to operate independently of the cropping scenario using a mixture of computer vision and ML techniques. To harvest strawberries, Kirk et al. (2020) used a Thorvald robot (Grimstad and From, 2017) with an RGB-D camera and on-board computing with AI components. This technique was able to localize and harvest strawberries with high accuracy using an automatically controlled specialized gripper. Sweeper (Arad et al., 2020) is a robotic platform, built on a lifting trolley with a harvesting arm at the front, capable of harvesting sweet pepper in a commercial greenhouse setting. Their traditional ML technique was able to accurately segment the fruit and detect the stem for harvesting. Despite the intertwined nature of robotic platforms and their algorithms, robotic vision algorithms are becoming more general.

### 2.2. Vision in Agriculture

In the previous decade, multiple specialized vision algorithms were developed for detecting crops. An early example of this was the work for grapes in a vineyard by Nuske et al. (2011, 2014) who primarily concentrated on predicting yield. To detect the key points of grapes, they explored radial symmetry and their own novel maximal point detection algorithm, along with investigating several other tradition ML and computer vision approaches. Interestingly, these techniques were developed to reduce human impact when surveying the fields. Similarly, while investigating yield, Wang et al. (2012) used stereo cameras at night with controlled artificial light to count apples. Their approach employed traditional computer vision techniques including detection in the HSV space. For detection and yield estimates of tomatoes, Yamamoto et al. (2014) also collected data at night with a known light source. They segmented the tomatoes using a pixel-wise decision tree which they trained using channels from five different color spaces. Overall, they achieved impressive results, particularly for mature (red) tomatoes. Hung et al. (2013) proposed a yield estimation approach for almonds and achieved impressive performance using a combination of a sparse auto-encoder and conditional random fields (CRF). Using a similar pipeline to Hung et al. (2013) and McCool et al. (2016) exploited these techniques for one of the earliest techniques for sweet pepper predictions. Their approach was able to achieve results similar to a human. Object (sweet pepper, tomatoes, and almonds) segmentation is an important component in many state-of-the-art robotic platforms for a number of tasks. Early works such as Nuske et al. (2011) and Hung et al. (2013) aimed to exploit various ML techniques to semantically locate and classify small objects, grapes, or almonds. However, it was noted that these techniques were heavily impacted by occlusions which degraded performance. In an effort to alleviate these occlusion issues, Zabawa et al. (2019) turned a two class problem into a three class segmentation task by incorporating edge information and utilizing deep learning methods.

These methods, excluding Zabawa et al. (2019), all relied on traditional computer vision approaches where hand crafted features were defined for each crop, however, more recently, deep learning approaches, such as DeepFruits, have been proposed by Sa et al. (2016). Such approaches are amenable to being deployed to multiple crops. These, more generalizable, approaches are integral if we are to perform automated monitoring without human intervention.

In recent years, deep learning based approaches are becoming more prevalent due to their accuracy and diversity from classification to segmentation. These are generally data driven approaches that rely heavily on labeled inputs at training time, which drives both a learned feature space and impressive results. Sa et al. (2016) was one of the first to apply Faster region-based convolutional neural network (Faster-RCNN) (Ren et al., 2015) for fruit detection. Koirala et al. (2019) and Tian et al. (2019) compared Faster-RCNN and Yolo (Redmon et al., 2016) for mango and apple detection, respectively, with Yolo capable of real-time performance with high levels of accuracy. Wan and Goudos (2020) proposed methods to speed up Faster-RCNN for fruit detection and achieved similar speed to Yolo-v3 (Redmon and Farhadi, 2018) while obtaining higher accuracy. Again for fruit detection in an orchard, Zhang et al. (2019) used a multi-task cascaded convolutional network and showed that network fusion had benefits for detection, however, the cascaded network structure added system complexity. Bargoti and Underwood (2017) outlined the benefits of convolutional neural networks (CNN) for fruit segmentation. More recently, the potential to perform fruit detection in the wild was explored by Halstead et al. (2020). It was shown that impressive performance for fruit detection could be achieved in vastly different fields by leveraging multi-task learning. In general, these approaches concentrate on detection, however, for yield estimation tracking approaches are necessary to ensure objects are only counted once.

### 2.3. Object Tracking in Agriculture

Tracking techniques have vast applications from simple particle filters for pedestrians (Denman et al., 2015) to precision agriculture from UAVs (López et al., 2021). These techniques have varying complexity and often rely on intricate hyper-parameter tuning or require innate knowledge about the network being used (Wang et al., 2019).

For tracking sweet pepper in agriculture, Halstead et al. (2018) and Smitt et al. (2021) make similar assumptions. As a robotic platform traverses a row, the scene between two consecutive images can be considered static in nature both spatially and temporally (i.e., objects in the image are spatially similar). These assumptions depend heavily on the frames per second (fps) of the camera and the velocity of the platform, if the fps is low or velocity is high this creates larger displacement between consecutive images and the assumption of a static scene no longer holds. Halstead et al. (2018) exploits this static scene for detection, relying heavily on the overlap between objects at *t* and the same object at *t* + 1. Smitt et al. (2021) extends this by creating a more accurate representation of an object in frame *t* at frame *t* + 1 using reprojection based on the wheel odometry. This approach was also able to reconcile larger distances between frames (i.e., *t* to *t* + *N*) creating a more robust and accurate tracking approach. While there are significantly more complex tracking algorithms (Jayaraman and Grauman, 2016; Stein et al., 2016; Wang et al., 2019) in our data, we are able to assume and leverage this spatial and temporal consistency.

More recently, robotic platforms that better integrate vision systems, deep learning, and robotics have been proposed. These robots can perform not only a specific action or intervention but also repeatedly estimate the state of the field. An example of this is PATHoBot (Smitt et al., 2021) which we proposed to combine advances in deep learning with robot (platform) information to provide a more robust crop monitoring approach. In this approach, the stereo data and wheel odometry was utilized to improve a tracking algorithm by reprojecting the masks detected by a deep learned model. However, the proposed robotic vision algorithm was applied on a single robot (PATHoBot) and to a single crop (sweet pepper).

We greatly extend our prior work (Smitt et al., 2021) and demonstrate the potential of the algorithms to be crop and robotic platform agnostic. For this, we propose and evaluate extensions to the reprojection for tracking. This is employed on two robots, PATHoBot (Smitt et al., 2021) and BonnBot-I (Ahmadi et al., 2021a), in both an arable farming and horticultural setting.

## 3. Materials

**Figure 2:**
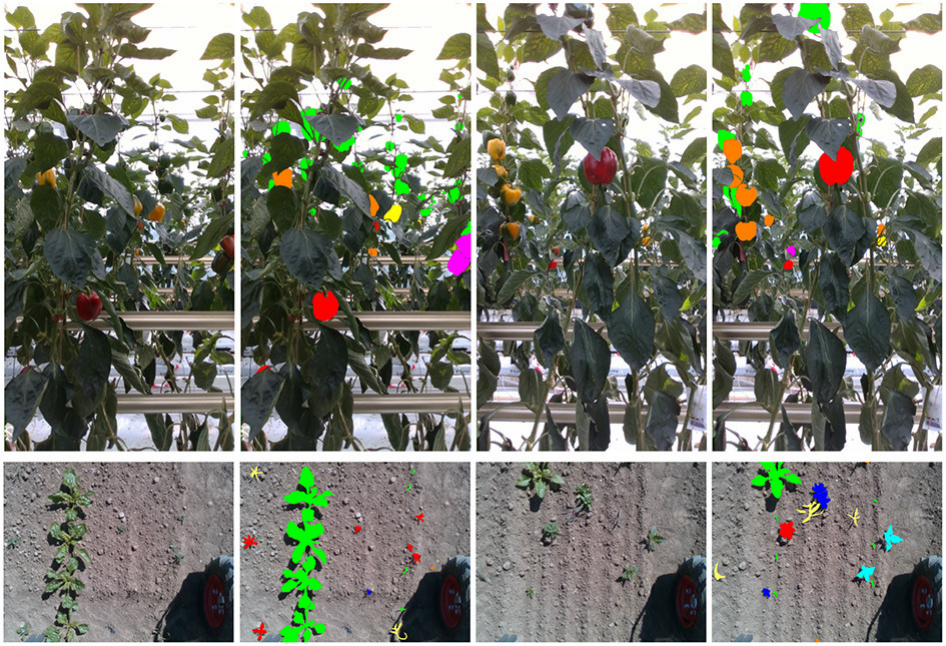
Two examples (one RGB and one ground truth mask for each example) from each of the two datasets. **Top row** outlines the sweet pepper dataset (BUP20) where the colors represent the sub-class ripeness labels. **Bottom row** shows the sugar beet dataset (SB20) where the colored masks represent the plant species.

In this article, we extend our prior work on PATHoBot (Smitt et al., 2021) to develop a platform and environment agnostic monitoring algorithm. The algorithm is deployed on a glasshouse robot (PATHoBot) and an arable farming robot (BonnBot-I), as shown in Figure 1. For each robot, we have collected a dataset and example images that are presented in Figure 2. The data for PATHoBot is used for the monitoring of sweet pepper (BUP20) and BonnBot-I is used for monitoring sugar beet and the associated weeds (SB20).

There are two critical aspects to these datasets. First, they have labeled instance segmentation masks and full temporal sequences for tracking. Second, they have important robot and scene information, such as registered depth images, camera parameters, and wheel odometry information.

For training and evaluation of the instance segmentation algorithm, both datasets consist of non-overlapping annotated images. SB20 consists of 71, 37, and 35 images for the training, validation, and evaluation sets, respectively. BUP20 consists of 124, 63, and 93 images for the training, validation, and evaluation sets, respectively. Furthermore, a specific row is assigned only to training, validation, or evaluation to ensure there is no overlap. This allows us to track an entire row assigned to validation or evaluation without bias from the training data. The tracking set of four rows for the SB20 dataset contains between 1,151 and 1,525 images and the five rows for BUP20 have approximately 1,799 frames.

### 3.1. Sweet Pepper Dataset (BUP20)

The sweet pepper dataset was captured at the University of Bonn's campus Klein-Altendorf (CKA) in a commercial glasshouse. The dataset was captured by Smitt et al. (2021) on PATHoBot under similar conditions to that captured by Halstead et al. (2020). Figure 2 (top row) outlines two examples of the RGB and masks from the BUP20 dataset. We use the same images as Smitt et al. (2021), which were captured on an Intel RealSense 435i camera (Intel Corporation, Santa Clara, California, USA.) (with a resolution of 1280 × 720), for evaluation purposes.

The BUP20 dataset captured two different cultivars: *Mavera* (green-yellow) and *Allrounder* (green-red). A breakdown of the training, validation, and evaluation sub-classes (ripeness/quality) can be seen in Table 1. While green dominates, there is still a rich representation of all the sub-classes.

**Table 1:** The training, validation, and evaluation breakdown of the two datasets, the sweet pepper dataset (BUP20) and the sugar beet dataset (SB20).

**Name**	**Abbreviation**	**Train**	**Validation**	**Evaluation**
**BUP20**				
Red	Rd	158	52	100
Yellow	Yl	318	98	181
Green	Gn	2774	1285	1466
Mixed Red	Mr	100	62	70
Mixed Yellow	My	189	101	143
**SB20**				
Sugar Beet	SB	388	151	231
Chenopodium Album	Ch	106	37	89
Thlaspi Arvense	Th	317	392	67
Fallopia Convolvulus	Bi	166	64	16
Persicaria Lapathifolia	Pe	313	171	139
Unknown	Uk	116	58	37
Chenopodiastrum Hybridum	Cy	10	7	47
Anthemis Arvensis	An	8	8	3

To quantitatively evaluate the performance of the tracking algorithms, we performed further annotation of the data. Using the available video sequences, three annotators visually counted the presence of the five sub-classes in the image sequences and ensured that each sweet pepper was only counted one time. This provided us with the ground truth data with counts for each sub-class over an entire row. These results are summarized in Table 2, where we calculate the average count of the three annotators for each sub-class then rounded up to the nearest integer. BUP20 was a complicated dataset to annotate as fruit could appear in the image from distant rows. Annotators were instructed to use the heat rails, which are approximately 1.05 m from the sensor, as a guide; if fruit appeared beyond this point, it was not counted. This interpretation of fruit location along with juvenile peppers appearing similar to leaves and varying levels of occlusion lead to some ambiguity in the annotations.

**Table 2:** The yield counts based on the average and rounded values from three annotators for the BUP20 dataset across the validation and evaluation rows.

**Row**	**Red**	**Yellow**	**Green**	**Mixed Red**	**Mixed Yellow**	**Total**
24-R4	10	17	212	6	15	260
24-R5	10	6	157	8	21	202
01-R4	13	24	231	9	15	292
01-R5	6	26	158	4	7	201
01-R6	11	13	192	11	12	239

### 3.2. Sugar Beet Dataset (SB20)

The sugar beet 2020 dataset was captured using BonnBot-I (Ahmadi et al., 2021a) also at CKA. BonnBot-I is a modified Thorvald robotic platform and the data was captured using a downward facing Intel RealSense D435i camera (Intel Corporation, Santa Clara, California, USA). The D435i provides RGB and registered depth while BonnBot-I provides wheel odometry. Together this makes it ideal for segmentation and tracking.

The sugar beet 2020 dataset, as shown in Figure 2 (bottom row) for example, is a challenging dataset created for weed classification and segmentation purposes with a resolution of 480 × 640. Plants are labeled into seven species plus an unknown label as outlined in Table 1, along with their fine-grained location. The unknown class is used for samples that are too small to classify or where there is high uncertainty about the species. The SB in this dataset primarily range from early youth (two seed leaves) to late youth stage (up to four foliage pairs) as described by Meier (1997), however, outliers exist. A key challenge of this dataset is the large difference in sample numbers, in the training set, there are 388 SB samples while *Chenopodiastrum hybridum* (10) and *Anthemis arvensis* (8) contain significantly less.

Similar to BUP20, the video sequences were analyzed to provide a summary of the number of plants present in the field. Three annotators counted the presence of the sub-classes in the sequences and the counts were then averaged over the three annotators; the average count value was rounded up to the nearest integer. A summary of the visual ground truth number of plants in the field is provided in Table 3 where it can be seen that the species' distribution is representative of pixel-level annotations in Table 1 (i.e., poor sample distribution of some classes). Compared to BUP20, the ground plane significantly reduced annotation complexity as plants could not appear beyond this point.

**Table 3:** The yield counts based on the average and rounded values from three annotators for the SB20 dataset across the validation and evaluation rows.

**Row**	**SB**	**Ch**	**Th**	**Bi**	**Pe**	**Uk**	**Cy**	**An**	**Total**
R1	120	16	185	49	80	43	6	5	504
R3-03	93	45	207	36	159	20	6	3	569
R3-09	167	16	12	14	15	24	1	1	250
R11	128	60	33	18	131	24	47	3	444

### 3.3. Evaluation Measures

For the task of object detection, we employ the F1 metric, which summarizes the precision-recall curve into a single value. For semantic segmentation, we use the intersection over union (IoU) and for classification, we use confusion matrices with an average accuracy score (conf_*acc*_). Finally, for tracking, we use the coefficient of determination (*R*^2^) and the mean normalized absolute error (μ*NAE*). We provide more details on each of these below.

The precision-recall curve describes the performance of a two-class classifier (e.g., object detector) and can be summarized by the *F*_1_ score. The precision $P=\frac{T_{P}}{T_{P}+F_{P}}$ and recall $R=\frac{T_{P}}{T_{P}+F_{N}}$ are defined by *T*_*P*_ which is the number of true positives (correct detections), *F*_*P*_ which is the number of false positives (false detections), and *F*_*N*_ which is the number of false negatives (miss detections). The value for *P* and *R* will vary as the threshold for the classifier varies and to summarize the resultant curve, we calculate the *F*_1_ score. This score is the point at which the precision equals the recall,


(1) $$\begin{array}{l} F_{1}=2\times \frac{\text{P }\cdot \text{ R}}{\text{P}+\text{R}}. \end{array}$$

The IoU metric describes the accuracy of semantic segmentation. Given the output of a system *O* and the ground truth *GT*, the IoU is given by


(2) $$\begin{array}{l} \text{IoU }\left(O,GT\right)=\frac{O\cap GT}{O\cup GT}. \end{array}$$

The maximum IoU is 1.0, which indicates perfect semantic segmentation.

For sub-class performance, we also calculate the average accuracy based on the confusion matrix, such that


(3) $$\begin{array}{l} {\text{conf}}_{acc}=\frac{1}{I}\overset{I}{\underset{i}{\sum }}C_{ii}, \end{array}$$

where *C* is a normalized *I* × *I* confusion matrix and the accuracy is calculated by summing the diagonals and dividing by the number of rows. This provides the average accuracy of the confusion matrix where a value closer to 1.0 indicates the higher performance.

Finally, for our tracking analysis, we utilize two metrics, the coefficient of determination (*R*^2^), and the mean normalized absolute error (μ*NAE*),


(4) $$\begin{array}{l} \mu NAE=\frac{1}{I}\overset{I}{\underset{i}{\sum }}\frac{\left|GT_{i}-P_{i}\right|}{GT_{i}} \end{array}$$

where *I* is the number of rows being evaluated, *GT* is the ground truth, and *P* is the predicted count. These results are calculated on the total number of objects counted against the ground truth to allow direct comparison to Smitt et al. (2021). It should be noted that this metric has a lower bound of zero (our desired outcome) but it is unbounded in the opposite direction. This is due to the prediction being scaled by the ground truth; if the prediction is significantly higher than the ground truth, this value can exceed 1.

## 4. Methods

We propose extensions to our prior work (Smitt et al., 2021) and demonstrate that the algorithms are both crop and robotic platform agnostic. Figure 1 provides a general overview of our monitoring method. For this, we propose and evaluate extensions to the reprojection for tracking and deploy them on PATHoBot and BonnBot-I in a horticultural and an arable farming setting, respectively.

The robotic vision pipeline is identical for both robots, only the deep learned model varies (with varying sub-classes). First, the robot scans the row, segments the desired objects, and then calculates their individual areas. The next step uses tracklets to aggregate the segmented data using a tracking-*via*-segmentation approach. Finally, the tracklet information is interpreted to supply a final yield and maximum area estimation.

### 4.1. Instance-Based Semantic Segmentation

The accurate localization of objects in the scene plays a key role in the overall impact of this technique. To achieve this, we utilize instance segmentation masks from Mask region-based convolutional neural network (Mask-RCNN) (He et al., 2017) as the base network. In its standard form, Mask-RCNN is able to provide classification scores (of *N* classes), bounded regions, and instance masks.

**Figure 3:**
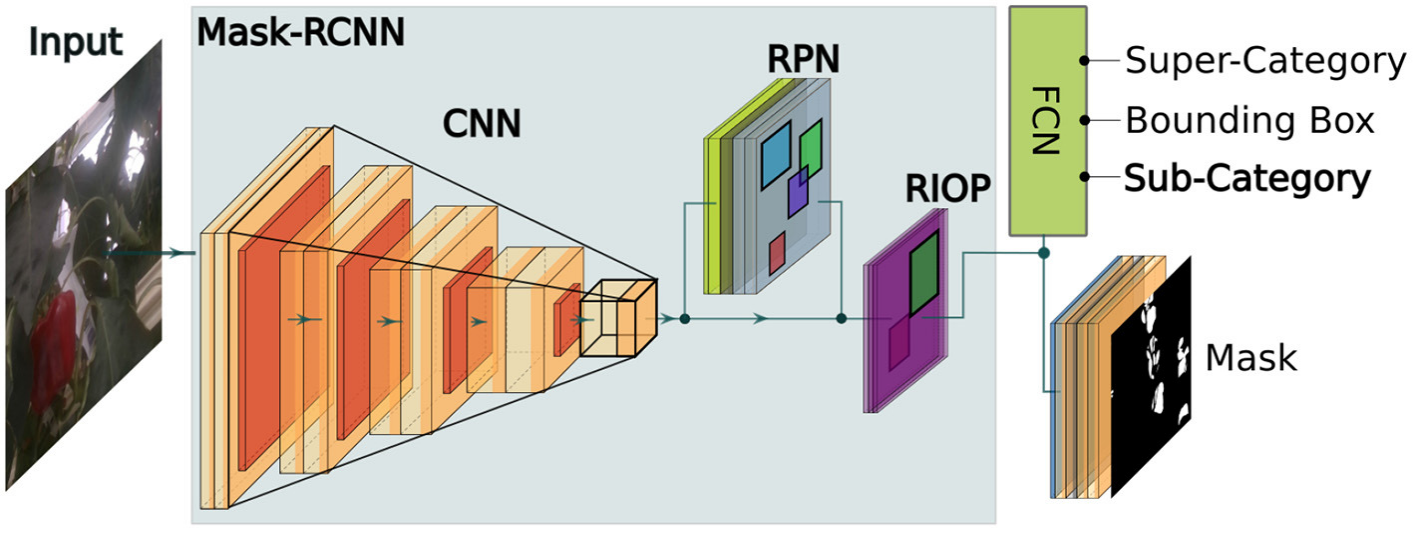
An overview of the Mask-RCNN network with the parallel sub-class classification layer included to calculate the quality (ripeness) of sweet pepper in the glasshouse or the species of crops/weeds in arable farmland.

We enhance our previous work in Halstead et al. (2018, 2020), where the quality (ripeness) was introduced as a parallel classification layer, by extending it to an arable field setting. Our work in Halstead et al. (2018) clearly describes the network architecture and evaluates its performance. Figure 3 outlines the proposed super-class (category) and sub-class (category) layout for both the arable farmland and glasshouse. This fully connected parallel layer is added after the final embedding layer of the network. The super-class represents either plants (SB20) or fruit (BUP20) and the sub-class represents the *N* finer-grained classes. For SB20, *N* = 8 species of plants and for BUP20, *N* = 5 ripeness estimates (red, yellow, green, mixed-red, and mixed-yellow). We use this approach as our previous work (Halstead et al., 2018, 2020) demonstrated that introducing a single super-class provided superior performance for the detection of sweet pepper (compared to *N* super-classes). This was attributed to the fact that all samples could be used in a single network structure to derive a strong generalized super-class classifier to detect the presence of sweet pepper (BUP20). The benefit of our approach is that we maintain super-class generalizability while also performing fine-grained classification in the sub-class. In this article, we show that our network can also be derived for plants (SB20).

We implement our Mask-RCNN style network (with sub-class layer - see Figure 3) in PyTorch for 500 epochs and a learning rate of 0.001 using the stochastic gradient descent (SGD) optimizer. For BUP20, similar to Ahmadi et al. (2021b) we resize the images to 704 × 416 and use the full resolution of SB20 (480 × 640). A batch size of six is used during training and the validation set is used to select the best model for evaluation; this means a model can be selected earlier than the 500th epoch. These hyper-parameters are identical for each dataset where the only variation comes from the information and name of the super-class (plant and sweet pepper) and the number of classes for cross entropy loss in the sub-class layer (five for BUP20 and eight for SB20).

### 4.2. Estimating the Area

Section 4.1 outlined the instance segmentation component of this technique. For intervention decision, the quality or species of the object alone is not enough for truly informed decisions. Area estimation of the object provides an extra layer of information relevant to both weeding and harvesting. This allows for better decision for crop, labor force, and business management (i.e., numerous full size ripe sweet pepper - harvest).

To calculate the object area (plant or sweet pepper), we exploit the stereo vision ability of the sensors on both platforms. Once object segmentation is obtained (section 4.1), we calculate the area using the registered depth and camera focal length. The area (*A*) of the *m*th object is calculated such that,


(5) $$\begin{array}{l} A_{m}=\overset{N}{\underset{i}{\sum }}\frac{d_{i}^{2}}{f_{x}\times f_{y}} \end{array}$$

where *N* is the total number of pixels in the segmented region (1*D* vector) and the depth values (*d*- in meters) are taken from the registered depth image. Finally, *f*_*x*_ and *f*_*y*_ are the two focal length parameters of the camera, taken from the camera intrinsics.

This highlights the advantage of using an RGB-D sensor. By exploiting this sensor framework, we are able to provide richer information about the farm to the end user. No prior assumption to determine the scene scale is required, like height in Lüling et al. (2021), to directly extract depth (and consequently area) information of crop surfaces, since the employed sensors provide pixel-wise depth information.

### 4.3. Tracking-*via*-Segmentation

Tracking crop (objects) in a field is imperative if the aim is to count the yield. This ensures that double-counting of the crop is avoided. To achieve this, we propose a tracking-*via*-segmentation approach based on Halstead et al. (2018) and Smitt et al. (2021). Both approaches exploit the static nature of an agricultural scene as a robot traverses a row where over a short timeframe scenes remain relatively unchanged. Smitt et al. (2021) further expands this by incorporating the wheel odometry and depth images to reproject the masks of the objects at *t* into a subsequent frame at *t* + *N*. Reprojection in conjunction with the instance based segmentor creates a more robust matching framework between frames.

An overview of our IoU based approach is outlined in Algorithm 1. Initially, the algorithm requires three base parameters: γ as the IoU matching threshold between *tracklets* and new masks, α a parameter to allow missed detections, and an empty *tracklets* list. The *tracklets* form the baseline of this approach and are used to maintain the identity of an object (crop/fruit) and aggregate the instance segmentations as the robot traverses the scene. The algorithm starts by iterating over the sequence of images in an ordered manner. Our Mask-RCNN network with sub-classes (Θ) is used on the image to extract the mask and other relevant information (like sub-classes).

**Algorithm 1:** Tracking-via-segmentation algorithm.

γ, α, *tracklets*
**for** *img* ∈ *images* **do**
*masks* = Θ(*img*)
*m* = *masks* ⊂ *FOV*
**if** *tracklets* == *None* **then**
*tracklets* ← *m*
**else**
*IoU* ← Φ(*tracklets, m*)
**while** *max*(*IoU*) > γ **do**
*i* ← *index*(*max*(*IoU*))
*tracklets*[*i*](*m*[*i*])
*IoU*[*i*] = 0 ⊳ Ensures this set is not reused
**end while**
*tracklets* ← *Unused*(*m*)
Ψ(*tracklets*, α)
**end if**
**end for**

Mask-RCNN (Θ(.)) provides a set of masks which are then processed. First, these masks are compared to a field of view (FOV) operator to ensure the mask is fully visible within the image (initialization and exit zones per, Halstead et al., 2018). The masks from the current frame are compared to all existing tracklets in a greedy manner, where the operator Φ(*tracklets, m*) outputs a matrix of IoU values; when reprojection is used the last mask in the tracklet is reprojected to the current frame. If no *tracklets* exist, a new set is initialized based on the current set of masks.

The IoU matrix is used to match between the *tracklets* and new regions in *m*. To assign *tracklets* to new segmented regions in *m* we calculate the maximum IoU in the matrix, this generates a *tracklet* and *m* matched pair. If this IoU value is above γ the *tracklet* is updated with the assigned *m* information. To ensure this pair is removed from further consideration their associated IoU values are set to zero (below γ). This matching process continues until the maximum IoU value is below γ or all of the *tracklets* are exhausted. The unused *m* detections are used to create new *tracklets* while Ψ is used to turn off any existing *tracklets* that have not been updated for α frames.

The matching criteria in Φ is critical to the *tracklet* updating based on the new information *m*. We investigate four criteria: intersection-over-union (IoU) with and without reprojection and a novel dynamic radius with and without reprojection. The two primary approaches (IoU and dynamic radius) are described individually and then the reprojection which is common to both, when used, is outlined.

#### 4.3.1. IoU Threshold

The IoU thresholding technique relies heavily on the pixel and shape consistency between images. Halstead et al. (2018) used this technique successfully for detection where they relied on bounding boxes rather than segmented regions. This approach was investigated in Smitt et al. (2021) for a segmentation based approach, however, the unconstrained shape (compared to bounding boxes) was limiting. The IoU criterion compares all active tracklets at *t* − 1 to all new segmentation masks at *t*, such that,


(6) $$\begin{array}{l} \Phi \left(T,s\right)=\frac{T⋂s}{T⋃s}, \end{array}$$

where *T* indicates a tracklet and *s* is the segmented region from Mask-RCNN. This criterion is then used to match tracklets to segmented regions based on the lower bound threshold γ (i.e., low IoU values are not matched).

**Figure 4:**
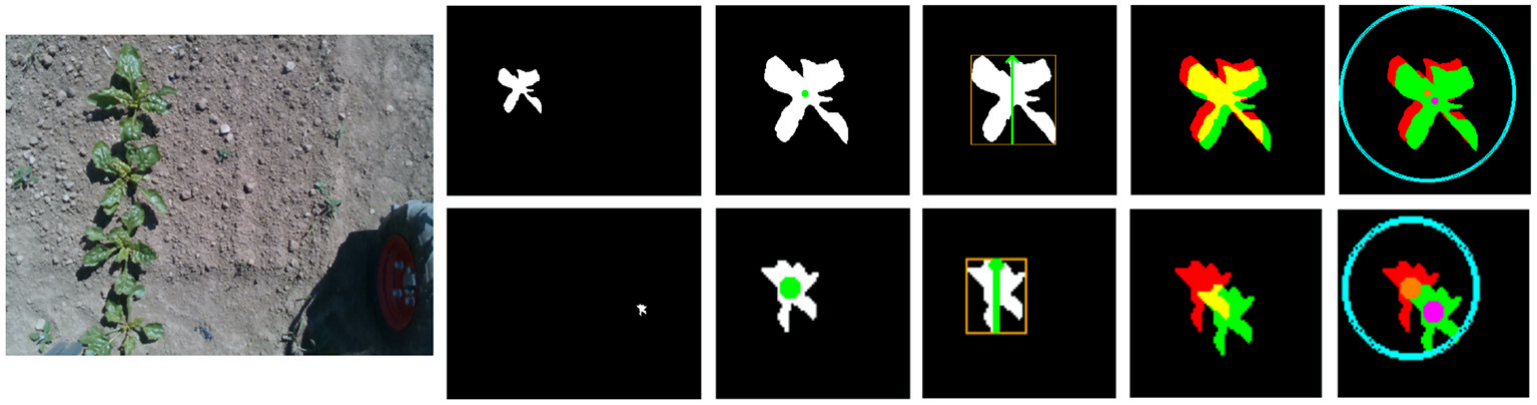
The dynamic radius calculation, far left is the original RGB image where we select two of the plants in the scene. **Top row** is a large crop example and the bottom row is a small weed. From left to right (after the RGB) the segmentation map, finding the center of mass location, calculating the radius of the search, the IoU after a small shift (5 pixels in each direction), and finally the centroid locations and the search radius. **Bottom row** has been scaled up in resolution to match the top row and is in fact a significantly smaller plant.

The primary issue with the IoU metric is that small shifts can greatly impact this score. This is particularly pronounced for small objects where, due to their size, small shifts can lead to disproportionately large changes in the IoU metric (see Figure 4). For this reason we explore a novel approach which we call dynamic radius.

#### 4.3.2. Dynamic Radius

The dynamic radius (DR) aims to overcome the limitations of a pixel-wise IoU approach. This is achieved by representing each object as a center point with a radius that is proportional to its size. Such an approach no longer relies on precise alignment which is central to the IoU approach. A visual comparison between DR and IoU is provided in Figure 4 with both small (bottom row) and large (top row) objects; the smaller plant is zoomed in further for visualization. The final two images of Figure 4 show the matching potential of DR with a small shift of five pixels in each direction. The IoU version (second from the right) has minimal overlap in the smaller plant, while the DR (right most image) still easily reconciles the new location within the matching region. Furthermore, even in the larger example, the unconstrained nature of the shapes makes it more difficult to match based on the IoU criterion.

The DR approach consists of the following steps. The center point is calculated directly as the center of mass (i.e., mean of *x* samples and mean of *y* samples). Next, we calculate the DR as the greatest distance based on the bounding box in either the *x* or *y* direction (Figure 4 - the third set of images from the right). This DR is then used to filter (i.e., cannot be matched) objects outside of this radius value using a Euclidean distance. For the tracking approach, the center of mass of a mask which is located closest to the tracklet (and inside the DR) will be matched.

#### 4.3.3. Reprojection

In both the IoU and DR metrics, there is a strong reliance on limited spatial shifts between frames and this can be confounded by several factors. For example, if the spatial shift is large enough, then the objects will not be tracked or if there is misalignment between the tracklets (between *t* − 1 and *t*), new objects can be instantiated. To alleviate some of these issues, Smitt et al. (2021) proposed to use reprojection so that the tracklets from *t* − 1 would be better aligned to the new segmented instances at *t*. This reprojection technique was able to increase tracking-*via*-segmentation performance in sweet pepper scenes.

To reproject a segmented tracklet mask from the previous frame *i* to the current frame *j*, the wheel odometry information is used. We calculate the camera homogeneous transform (**H**_*ij*_), such that,


(7) $$\begin{array}{l} {\text{H}}_{ij}={\text{E}}^{-1}{\text{W}}_{ij}\text{E}, \end{array}$$

where **W** and **E** represent the wheel odometry transform and camera extrinsics to the odometry frame, respectively. Now pixel coordinates **m** at frame *i* belonging to a detection mask $M_{i}=\left[{\text{m}}_{i1},{\text{m}}_{i2},\ldots ,{\text{m}}_{iN}\right]$ can be reprojected to frame *j* with,


(8) $$\begin{array}{l} {\text{m}}_{jk}=\pi \left({\text{H}}_{ij}\left(\pi^{-1}\left({\text{m}}_{ik},d_{m_{ik}}\right)\right)\right), \end{array}$$

where *k* = [1, …, *N*], π(.) is the camera projection function, *d*_*m*_ is each mask coordinate's depth value, and **H**(.) applies a homogeneous transform between frames.

The appearance of sweet pepper in distant rows is an additional complicating factor in BUP20. Depth filtering was found to help the tracking algorithm in Smitt et al. (2021). This ensures that only objects in the current row are tracked, regardless of the segmentation output. To do this, we count the number of pixels in the segmentation mask that fall between a lower depth threshold (τ_*l*_) and an upper depth threshold (τ_*h*_), such that,


(9) $$\begin{array}{l} q=\frac{1}{P}\overset{P}{\underset{i}{\sum }}g\left(d_{i}\right)\times 100, \end{array}$$

which returns *q* as a percentage of depth pixels within the range τ_*l*_ and τ_*h*_. In this case, *P* represents the total number of pixels in the segmented region and *d* represents the registered depth values associated with that region. The function *g*(.) returns a one if *d*_*i*_ is within τ_*l*_ and τ*h* else it returns zero. The value *q* can then be compared to a static threshold indicating whether the region falls within the filtered depth range.

## 5. Evaluation and Discussion

Our platform agnostic agricultural monitoring approach is deployed on two robots, PATHoBot and BonnBot-I, for horticultural and arable farming systems, respectively. We perform extensive evaluations for each component of our proposed approach. First, we evaluate the performance of the instance-based semantic segmentation algorithm. This includes the performance of crop detection as well as instance-based segmentation and sub-class classification accuracy. Second, the performance of the tracking approaches with and without reprojection are explored. We highlight the robustness of the reprojection approach by analysis the impact of large skips between subsequent frames. Third, a qualitative analysis of the results for two considerably different crops (sweet pepper and SB) is presented to highlight the potential of our approach.

### 5.1. Instance-Based Semantic Segmentation

Instance based segmentation forms the backbone of our proposed approach. We use Mask-RCNN and add a parallel layer for sub-class classification. The parallel structure ensures both general (super) and specific (sub) classification information is learned. Halstead et al. (2018, 2020) showed that using this parallel layer had considerable advantages when applied to sweet pepper detection (super-class) and ripeness estimation (sub-class). We demonstrate, for the first time, that this parallel layer can also be applied to plants in the field to perform plant detection (super-class) and species (sub-class) classification. Our models are evaluated in three steps: first, how well we detect objects in the scene; second, how well do we segment objects; and third, how accurate is our sub-class layer.

#### 5.1.1. Object Detection

**Figure 5:**
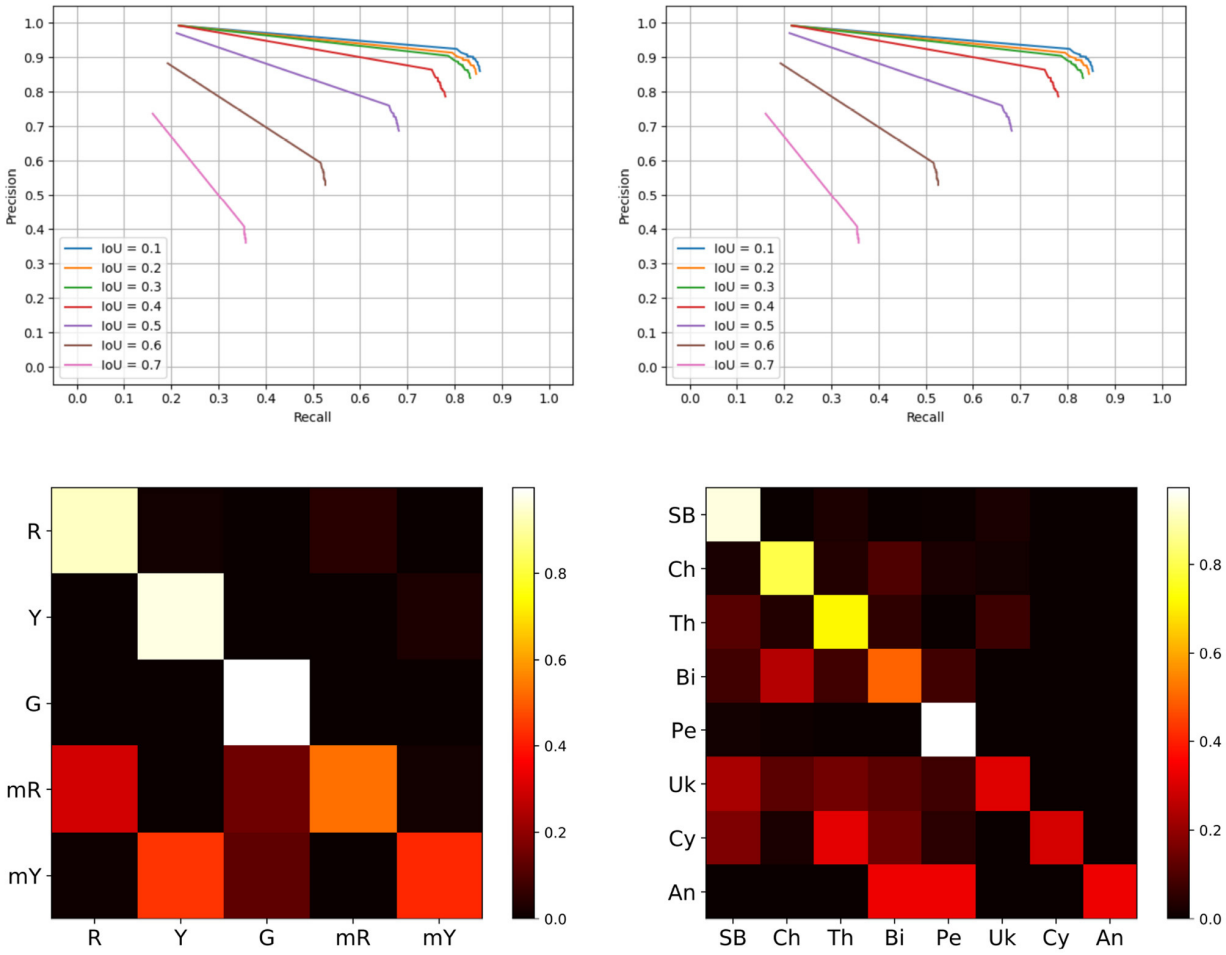
Evaluation results. **Top row** is the precision recall curves of the detection only system, left hand side is the BUP20 and right is SB20. **Bottom row** outlines the sub-class confusion matrix results based on the extended Mask-RCNN network, left is the BUP20 dataset, and right is SB20.

Object detection is a common metric for evaluating localization techniques. If the detected object overlaps sufficiently with its ground truth position, then it is considered to be a true detection, *T*_*P*_. The top row of Figure 5 (BUP20 left and SB20 right) outlines detection performance at different IoU values across the two datasets.

From this figure, it is evident that IoU values up to 0.4 perform well with noticeable degradation beyond. For system performance, we evaluate at an IoU of 0.4 as this reflects high performance and also ensures considerable overlap between the ground truth and the prediction. For the SB20 dataset, we are able to achieve a precision, recall, and F1-score of 0.865, 0.752, and 0.804 for plant detection alone. The BUP20 accuracy is somewhat less with scores of 0.783, 0.638, and 0.703 for precision, recall, and F1-score respectively, although the F1-Score was commensurate with Halstead et al. (2020) who achieved 0.762 on a similar sweet pepper dataset. We attribute this performance difference to the fact that BUP20 has high levels of occlusion and a more complicated scene with crop present across multiple depths (both near and far), as can be seen in the top row of Figure 2. By comparison, SB20 has a relatively easy to detect background with a simpler scene structure, as shown in the bottom row of Figure 2. For both environments, the result of object detection can be considered to be quite promising for extracting useful in-field information.

#### 5.1.2. Instance Segmentation

Instance segmentation relates not only if an object is detected (found) but also if all of the pixels relating to that object are found. For matching a ground truth and segment pair, similar to Halstead et al. (2018, 2020), we use an IoU threshold of 0.4. This threshold also outlined the best trade-off between overlap and accuracy from Figure 5. Once a ground truth and detection pair are matched using this threshold, we use the segmented pixel-wise IoU to determine performance. We report three metrics: background IoU (BG IoU), foreground IoU (FG IoU), and the average of the two μIoU. The BG IoU is the pixel-wise comparison between the ground truth background and the predicted background. Similarly, the FG IoU describes the super-class segmentation accuracy (ground truth to prediction). This pixel-wise comparison provides a more complete evaluation of the systems, performance by directly comparing the output from Mask-RCNN with the ground truth masks. The segmentation results are displayed in Table 4.

**Table 4:** Instance and semantic segmentation results for the two datasets.

**Dataset**	**BG IoU**	**FG IoU**	**μIoU**
**Instance segmentation**			
SB20	0.999	0.498	0.748
BUP20	0.999	0.433	0.716
**Semantic segmentation**			
SB20	0.977	0.726	0.851
BUP20	0.986	0.718	0.852

*For comparison, the background IoU (BG IoU), foreground IoU (FG IoU), and the average IoU (μIoU) are displayed*.

The parallel layer based Mask-RCNN approach achieves interesting instance based segmentation performance. From Table 4, for SB20, we achieve a foreground IoU (FG IoU) performance of 0.498 and 0.433 for BUP20. This outlines the impact that false and missed detections have on instance based performance. If we neglect these two properties (false and missed detections), we achieve FG IoU values of 0.765 for SB20 and 0.754 for BUP20. This indicates, on a pixel-wise level, that when we accurately predict a region our segmentation performance is high. However, the presence of both missed and false detections deteriorates the performance considerably.

To further illustrate this, we consider the two class semantic segmentation problem using the same network output (i.e., plant vs. background or sweet pepper vs. background). To achieve semantic evaluation, we create a single binary ground truth map where any pixel assigned to an object is scored as the positive class. Similarly, for the prediction, we use the calculated instances and assign positive pixels to the semantic prediction mask. This creates two binary masks, one based on the ground truth instances and one based on the predicted instances.

This result is shown in Table 4 where the μ*IoU* is above 0.85 for both SB20 and BUP20. Furthermore, for semantic segmentation, the FG IoU performance is high and in fact, is similar to the instance-based segmentation results when missed and false detections are removed. This indicates two things. First, that the lower performance of the instance-based segmentation is due to errors from the detection module. Second, because the instance-based metric is an average of IoUs from instances (even false ones), this can give the impression that the quality of the pixel-wise segmentation is low. Therefore, this further analysis shows that even though the detection module can introduce errors the overall quality of the pixel-wise segmentation is high.

#### 5.1.3. Sub-class Accuracy

In our previous work (Halstead et al., 2018, 2020), we exploited the super-class and sub-class properties of sweet pepper. Generally, sweet pepper share similar features in terms of shape and reflection with only color differences. For the first time, we extend this to arable farmland to investigate its performance for classifying specific species of crop and weed (plants). This is a considerably more difficult problem as these species have different growth properties, including unconstrained shapes. In these confusion matrices, we only compare IoU (i.e., above 0.4) matches between the ground truth and predictions which removes the impact of false positives (*F*_*P*_) and missed detections (*F*_*N*_). While both *F*_*P*_ and *F*_*N*_ play an important role in the overall accuracy of the system, the previous sections outline the limitations of the detection routine. The metric displayed in Figure 5 (bottom row) outlines our sub-class performance when we accurately detect an object.

Figure 5 (bottom right) outlines the sub-class performance for plant species classification using a confusion matrix. Overall, we achieve a confusion matrix average accuracy of 0.619. *Chenopodiastrum hybridum* (Cy) and *Anthemis arvensis* (An) are the worst performing species with accuracies of 0.256 and 0.333, respectively. Overall, we can attribute this low accuracy to the lack of samples both in the training (8 and 10) and evaluation (47 and 3) sets. By contrast, the species with a higher number of training samples achieve higher accuracy. For instance, SB and *Persicaria lapathifolia* (Pe) which have 388 and 313 training samples achieve accuracies of 0.930 and 0.964, respectively. The high accuracy for SB is of particular importance as both crop monitoring and precision weeding are primarily concerned with identfying the crop (in this case SB) and thus misclassifying weeds is less detrimental.

For the under represented sub-classes, data collection and annotation was a considerable bottleneck. To create a more even distribution, the weeds need to be present in the field, and this was not the case for the SB20 dataset. To alleviate the impact on a trained model, two key possibilities exist that could be explored in the future. First, data augmentation has been shown to improve networks while maintaining a small sample size by reproducing the same images with small augmentations. This approach could be considered in the future, however, it does not solve the skewed distribution of the dataset. Second, the data can be balanced through a weighting scheme at the sub-class classification layer. Weighting can create a more robust classification by focusing on accuracy for under represented classes creating a more even distribution.

For the BUP20 results, we refer to the confusion matrix on the left hand side of Figure 5 (bottom row). In this setting, we achieve an average accuracy of 0.772, which is considerably higher than SB20. We attribute this to the fact that color is a dominant factor and also to the fact that for SB20, there are two classes with a low number of samples. However, we do note that color is also a cause for confusability for BUP20. In particular, the mixed-red and mixed-yellow classes are often confused with full red and full yellow sweet pepper, respectively.

In summary, both SB20 and BUP20 were able to achieve promising performance for super-class and sub-class classification. This includes the novel environment of arable farmlands where species have significantly different visual properties. Overall, the pixel-wise object location along with the species (arable farmland) or ripeness (glasshouse) estimation is able to provide important additional phenotypic information to the farmer.

### 5.2. Tracking

Based on section 4.3, this section evaluates the performance of the tracker on both the BUP20 and in a novel environment SB20. From our previous work (Halstead et al., 2018; Smitt et al., 2021), we define the following hyper-parameters for the tracker. The keep running parameter which allows the tracklet to miss frames are set to 5. A minimum of 10 segmentation matches is required for a tracklet to be considered a valid track. For the IoU based criterion, we use a minimum threshold of 0.1 for reconciling tracklets to new regions. We also empirically evaluated values of 0.5, 0.75, and 1.0 for weighting the DR and found that a value of 1.0 was optimal.

#### 5.2.1. Sweet Pepper Tracking Evaluation

Table 5 outlines our BUP20 performance where we use the μ*NAE* metric described in section 3.3. This evaluation is performed over three maximum depth filtering values τ_*h*_ = [1.0, 1.4, *None*], and the minimum depth value τ_*l*_ is constant at 0.4 m. The value of 1.0 was selected as it approximated the distance from the heating rails to the sensors and 1.4 and *None* as a direct comparison to Smitt et al. (2021). For filtering out objects based on the depth we set a threshold such that *q* > 50, ensuring that at least 50% of the pixels appear in the depth range.

**Table 5:** BUP20 tracking results using the four criteria at three different depth filtering values, 1.0 m, 1.4 m, and no filtering.

**Approach**	**Depth 1.0**		**Depth 1.4**		**No filtering**	
	***R* ^2^**	***μNAE***	**R^2^**	***μ*NAE**	***R* ^2^**	***μ*NAE**
IoU	0.886	0.125	0.636	0.186	0.035	1.371
IoU Reproj	0.881	0.045	0.607	0.335	0.005	1.252
DR	0.897	0.039	0.673	0.294	0.015	1.743
DR Reproj	0.901	0.047	0.603	0.375	0.001	1.392

*Results display the R^2^ value and the mean normalized absolute error (μNAE)*.

These results reflect the annotation directive to not count objects appearing beyond the heat rails. From this, depth filtering plays a crucial role in obtaining accurate yield estimations. At a depth of 1.0, our best approach scored 0.039 compared to 1.252 for no depth filtering. While the no depth filtering score appears to indicate poor performance it more reflects the ability of Mask-RCNN to accurately reconcile small objects (such as those past the heat rails). From a segmentation perspective, the changes in illumination due to the position of the sun and platform had a minor impact, particularly in row six where the greatest illumination variation existed.

Overall, from Table 5, a depth filtering value of 1.0 achieved the best results, where even the worst performing approach (IoU only) scored 0.125. Interestingly at a depth of 1.0 m, the technique both under and over estimated the total yield across the four techniques, somewhat explaining the slightly low *R*^2^ values. While the DR approach scored similarly with and without reprojection its impact with IoU is clear improving the μ*NAE* score from 0.125 to 0.045. For DR, we attribute the reduced performance with reprojection to the limitations of the spatial criterion, it can match 360 degrees around the center mass.

#### 5.2.2. SB Tracking Evaluation

A benefit of the SB20 dataset is that the objects only appear on the ground plane, and there is little impact from the weather (both sun and wind). This enables a single tracking evaluation without depth filtering requirements, the results are displayed in Table 6. The high *R*^2^ values (all greater than 0.93) consistently explain the differences between our predictions and the ground truth using a linear model. Contrasting with BUP20, the SB20 yield consistently under counted, which could explain the higher *R*^2^ values. To better understand the performance of the different tracking criteria, we once again use the μ*NAE* score.

**Table 6:** SB tracking results using the four different criteria.

**Approach**	***R* ^2^**	***μNAE***
IoU	0.937	0.396
IoU Reproj	0.947	0.274
DR	0.957	0.214
DR Reproj	0.970	0.137

*Results display the R^2^ value and the mean normalized absolute error (μNAE)*.

Based on the μ*NAE* score, it can be seen that incorporating reprojection considerably improves performance. Incorporating reprojection into the IoU and DR criteria improves their absolute μ*NAE* score by 0.122 and 0.077, respectively. This is a relative improvement in estimating the number of plants present of 30.8 and 36.0% for IoU and DR, respectively. Also, in all cases, the DR approach outperforms the IoU approach (with or without reprojection). DR with reproejection achieves a score of 0.137, which is an improvement of 50% over IoU with reprojection.

#### 5.2.3. Tracking Over Large Skips

In the previous tracking evaluations, it was assumed that the frame rate was consistent. To fully analyze the reprojection performance, we evaluate the system performance when there is a skip of five frames between segmentation. This five frame skip explores the robustness of the approaches to inconsistencies such as faster or slower moving vehicles. In these experiments, we reduce the minimum tracks parameter to three due to the shorter amount of time objects remain in the scene. Table 7 clearly outlines the benefits of reprojection when the frame jump is large or inconsistent. The *R*^2^ value for the DR is high and misleading for two reasons, first it counts significantly less sweet pepper than actually exists. Second, due to the frame skip, tracklets reconcile with new objects passing through their stored region. For BUP20, the μ*NAE* value for IoU and DR both with reprojection scored 7.6 and 6.6%, respectively. This is considerable when contrasted to the standard IoU which has the worst performance at 73.1%. Overall, the ability to reconcile tracks over larger spatial distances, allowing for frame drops or faster vehicle motion, is a key benefit in using the reprojection based techniques.

**Table 7:** The impact of skipping five frames on the four different criteria in the BUP20 and SB20 datasets.

	**BUP20**		**SB20**	
**Approach**	***R* ^2^**	***μNAE***	***R* ^2^**	***μNAE***
IoU	0.221	0.731	0.950	0.633
IoU Reproj	0.823	0.076	0.870	0.499
DR	0.977	0.327	0.857	0.366
DR Reproj	0.776	0.066	0.960	0.329

#### 5.2.4. Current Limitations

Despite the impressive and robust performance that we have presented, there is a limitation with the proposed approach. The main limitation is the low performance with sub-class counting accuracy. For sugar beet, when we compare the total yield (or super-class) to species specific (sub-class) performance for DR reprojected, the μ*NAE* is degrades to 0.440 (from 0.137). Similarly for sweet pepper, for the best performing systems, the μ*NAE* for the sub-classes degrades from 0.039 to 0.35. This indicates that while we are able to achieve accurate localization, our fine-grained species classification, while promising, still requires improvement.

### 5.3. Qualitative Analysis

In parallel with our quantitative evaluations, we also perform two qualitative analyses. First, we evaluate the accuracy of our area estimation component. Second, we analyze the impact our monitoring technique could have on informed decision making.

#### 5.3.1. Area Estimation

**Figure 6:**
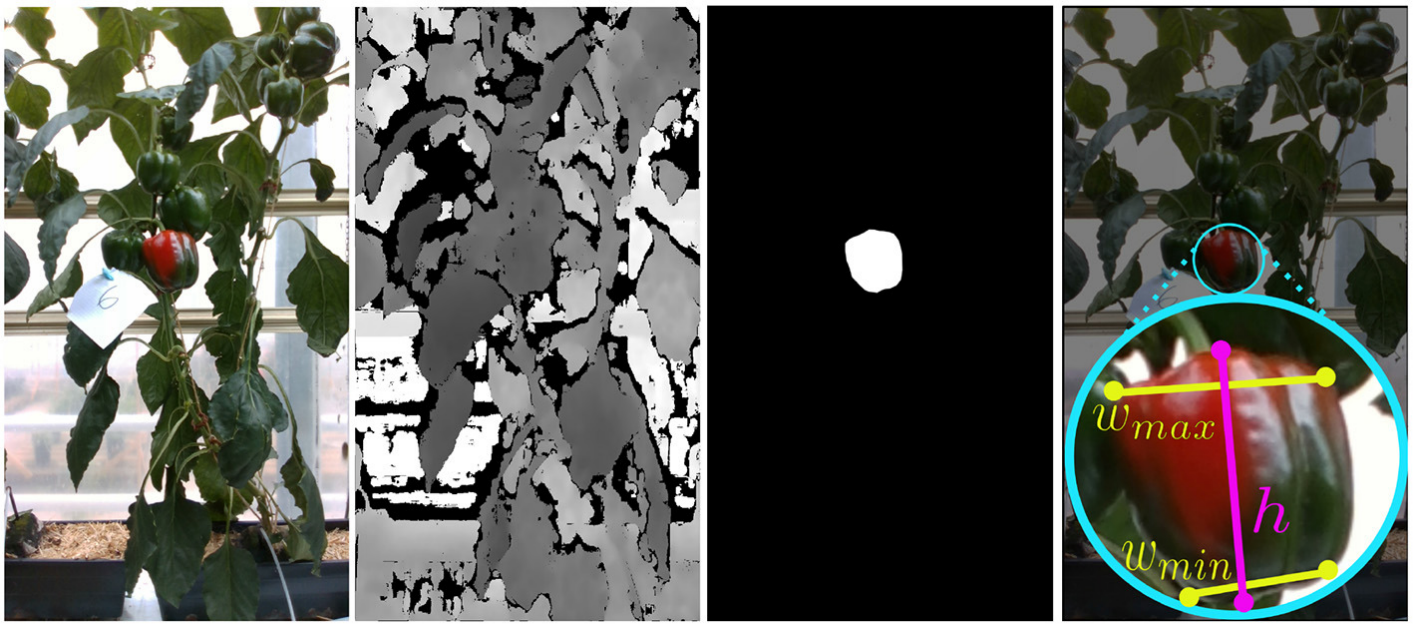
An example image from the area estimation analysis, from right to left, the original RGB image, depth for visualization, manual segmentation, and a visual approximation of the manual measuring points.

For a qualitative analysis of the area estimation, we coarsely measured then recorded ten sweet pepper on PATHoBot. Both the depth and RGB images were captured, and sweet pepper were manually segmented in the image. An example of these images with their annotations can be seen in Figure 6.

The coarse ground truth was measured with vernier calipers at the approximate positions shown in Figure 6. First, the maximum height (*h*) and width (*w*_*max*_) were measured, then, a value near the bottom of the sweet pepper (*w*_*min*_) that created a trapezoid shape were also measured. Using these distances, we create two area values for each sweet pepper, an upper bound of the sweet pepper area,


(10) $$\begin{array}{l} rec_{A}=h\times w_{max} \end{array}$$

and a lower area bound measured such that,


(11) $$\begin{array}{l} trp_{A}=rec_{A}-h\times \left(w_{max}-w_{min}\right) \end{array}$$

which creates the trapezoid shape. We assert that this is the lower bound of the object area as it does not include volumetric information about the object nor the rounded shape.

It can be seen from our results in Table 8 that our vision system consistently falls within the upper and lower bounds of the ground truth. This indicates that our vision only area estimation component is able to accurately define the seen area of the sweet pepper.

**Table 8:** Qualitative analysis of the area estimation of manually segmented sweet pepper.

**Sweet pepper**	***rec*_*A*_**	***trp*_*A*_**	**Visual**
0	76.8	47.2	66.4
1	75.4	44.3	70.5
2	85.5	58.2	66.6
3	81.2	47.0	72.0
4	80.2	48.6	76.3
5	81.9	55.8	71.3
6	81.9	53.4	72.0
7	86.7	56.1	71.5
8	90.2	65.3	76.1
9	84.6	57.9	76.6

*The visually calculated sweet pepper area should fall between the coarsely calculated upper bound (rec_A_) and lower bound (trp_A_). All values are in cm^2^*.

#### 5.3.2. Monitoring Algorithm

The individual components of our approach perform well on two different robots, one in arable farmland and the other in a glasshouse. The final qualitative analysis fuses these components into a single monitoring algorithm in both environments. We evaluate two rows from each environment with data captured a week apart, in between the captures, various crop management tasks were manually performed which augmented results. In all experiments, we used the DR with reprojection for tracking as it performed the best for SB20 and had a similar performance with the best in BUP20.

From the BUP20 dataset, captured on PATHoBot, we investigate two captures of rows four and five. We review this for both the sub-class yield and the area estimation. During this time periods some pruning and minor harvesting was completed by the staff.

Overall, harvestable yield counts went from 33 to 39 for row four and 24 to 38 for row five. Similarly, green sweet pepper broke to the mixed color with 243 to 236 (row four) and 184 to 151 (row five) resulting green peppers between the monitoring days. Interestingly, for green sweet pepper, for row four, the area increases from in 38.1 to 44 cm^2^, while row five stayed somewhat consistent 38.1 cm^2^ then 37.7 cm^2^. We attribute this to the almost ripe sweet pepper breaking color to mixed red/yellow and juvenile sweet pepper growing. This monitoring technique also provides marketing information; for row four, a total of 289 sweet pepper existed to potentially sell, compared to 220 for row five.

For the SB20 analysis, we choose data that was in the lowest controlled herbicide group (0–30%). This limited the intervention on the weeds ensuring they grew in size and amount. Overall, the average weed area grew for both row one and row eleven: 76 to 101 cm^2^, and 98.3 to 138 cm^2^. While there was not an increase in weed count for row one, row eleven increased from 292 to 351. Both of these values indicate the potential impact on crop growth as the weeds are growing which creates competition for soil nutrients.

**Figure 7:**
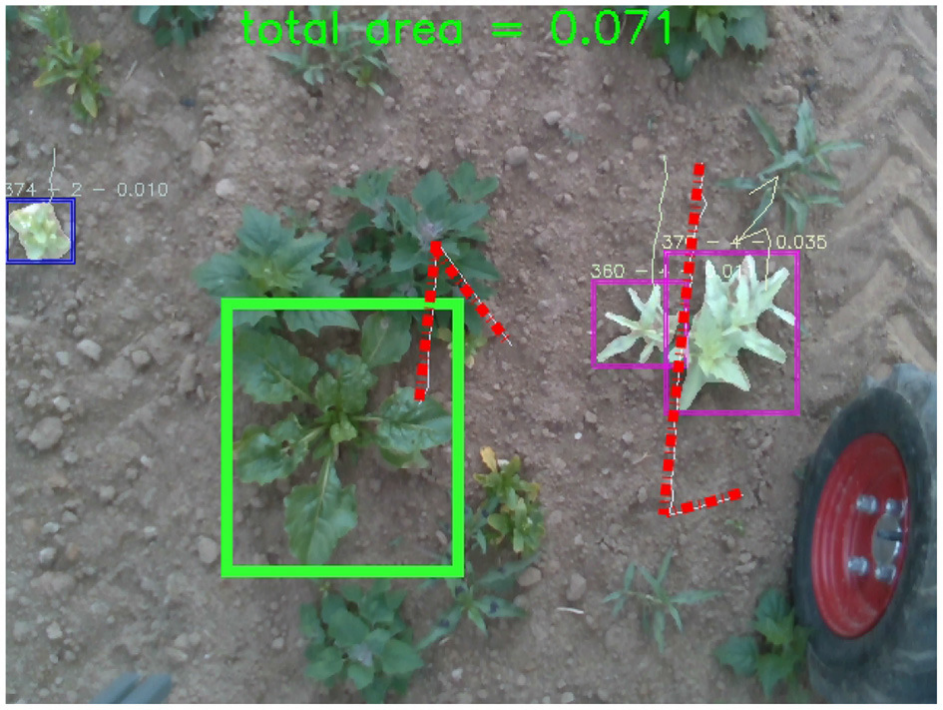
Examples of where the dynamic radius criterion fails. The green box indicates a completely missed SB plant, and the red dashed lines show how the criterion can change directions rapidly regardless of the direction of travel.

The impact of the weed growth, and the nature of the DR criterion, is outlined in the row, one crop yield estimate which reduces from 111 to 99. Figure 7 outlines two of the reasons for this drop in yield based on the DR criterion. The green bounding box indicates a missed detection when tracking, irrespective of Mask-RCNN accurately segmenting it. This is primarily due to the large objects surrounding it confusing the DR. Similarly, the red dashed lines outline a key limitation of DR, which matches based on the smallest Euclidean distance within the radius, regardless of the previous trajectory of the tracklet or the robot (i.e., it can match 360 degrees). While DR is generally a more accurate matching criterion, there are issues associated with the methodology, and future work can alleviate these.

Overall, this platform and environment agnostic monitoring algorithm provided important information to farmers to inform decisions. This approach also included marketing information about the crop or fruit yields and the impact of weeding paradigms on the crop in arable farmland.

## 6. Conclusion

In this article, we show that robotic monitoring algorithms can be designed to be platform and environment agnostic. We show that this deep learning approach, once data is provided, can be accurately segmented in either an arable farm or horticultural setting. Using the segmented output, we are also able to calculate phenotypic information in the form of plant size. This is a first step toward providing a summary of the state of the field.

Our crop agnostic monitoring algorithm extends a parallel classification structure in Mask-RCNN. This parallel structure was previously applied to sweet pepper in a glasshouse for crop detection (super-class) and ripeness estimation (sub-class). We show for the first time, that this parallel structure can be used to perform plant detection (super-class) and species-level classification (sub-class). This demonstrates the generalizability of our approach.

To accurately provide information to the farmer, we evaluated varying matching criteria for a tracking-*via*-segmentation approach. The key benefit of our novel dynamic radius with reprojection approach was its ability to match unstructured shapes more accurately than a pixel-matching based approach. While similar performance was achieved for sweet pepper, the strength of this spatial matching approach was seen in an arable farmland where the scenes were cluttered and the growth of objects unconstrained; we achieved a performance boost of 50% over the pixel dependent approach. This agnostic monitoring algorithm leveraged computer vision, deep learning, and robotics to reduce physical monitoring of fields by farmers. The fusion of these techniques provided raw information, such as the impact of weeding paradigms, to support intervention and management decisions.

## Data Availability Statement

The original contributions presented in the study are included in the article/supplementary materials, further inquiries can be directed to the corresponding author/s.

## Author Contributions

MH developed the concepts in the paper, designed the algorithm and experiments, and wrote the paper. AA and CS contributed to writing the paper and with experiments. OS contributed to writing the paper and gave a unique agricultural perspective. CM contributed to writing the paper and developing the concepts. All authors reviewed the results.

## Funding

This work has partially been funded by the Deutsche Forschungsgemeinschaft (DFG, German Research Foundation) under Germany's Excellence Strategy, EXC-2070 – 390732324 – PhenoRob.

## Conflict of Interest

The authors declare that the research was conducted in the absence of any commercial or financial relationships that could be construed as a potential conflict of interest.

## Publisher's Note

All claims expressed in this article are solely those of the authors and do not necessarily represent those of their affiliated organizations, or those of the publisher, the editors and the reviewers. Any product that may be evaluated in this article, or claim that may be made by its manufacturer, is not guaranteed or endorsed by the publisher.
